# Drift-Free Integration in Inductive Magnetic Field Measurements Achieved by Kalman Filtering

**DOI:** 10.3390/s22010182

**Published:** 2021-12-28

**Authors:** Pasquale Arpaia, Marco Buzio, Vincenzo Di Capua, Sabrina Grassini, Marco Parvis, Mariano Pentella

**Affiliations:** 1 Department of Electrical Engineering and Information Technology, University of Naples “Federico II”, 80100 Naples, Italy; vincenzo.di.capua@cern.ch; 2 Technology Department, European Organization for Nuclear Research (CERN), 1211 Geneva, Switzerland; marco.buzio@cern.ch (M.B.); mariano.pentella@cern.ch (M.P.); 3 Department of Applied Science and Technology, Polytechnic of Turin, 10129 Turin, Italy; sabrina.grassini@polito.it; 4 Department of Electronics and Telecommunications, Polytechnic of Turin, 10129 Turin, Italy; marco.parvis@polito.it

**Keywords:** drift-free integration, integration drift, sensing coils, magnets, magnetic measurements, sensor fusion, Hall probe, Kalman filtering

## Abstract

Sensing coils are inductive sensors commonly used to measure magnetic fields, such as those generated by electromagnets used in many kinds of industrial and scientific applications. Inductive sensors rely on integrating the output voltage at the coil’s terminals in order to obtain flux linkage, which may suffer from the magnification of low-frequency noise resulting in a drifting integrated signal. This article presents a method for the cancellation of integrator drift. The method is based on a first-order linear Kalman filter combining the data from the coil and a second sensor. Two case studies are presented. In the first one, the second sensor is a Hall probe, which senses the magnetic field directly. In a second case study, the magnet’s excitation current was used instead to provide a first-order approximation of the field. Experimental tests show that both approaches can reduce the measured field drift by three orders of magnitude. The Hall probe option guarantees, in addition, one order of magnitude better absolute accuracy than by using the excitation current.

## 1. Introduction

### 1.1. Problem Statement

Sensing coils, also called pick-up coils, are among the most commonly used sensors for measuring a time-changing magnetic field owing to their low cost, linear behavior, and easy calibration. Vast literature is available on their design, fabrication, characterization, and operation [1,2,3,4,5]. Sensing coils consist of a series of conducting loops linking the magnetic flux and exhibiting a voltage at their terminals proportional to the time derivative of the flux, according to Faraday’s law of induction. The average magnetic flux density over the coil can be calculated by integrating the voltage measured at the terminals, assuming that the input impedance of the Data Acquisition System (DAQ) is infinite:

(1) $$B\left(t\right)=B_{0}+\frac{1}{A_{c}}\int_{0}^{t}v\left(\tau \right)d\tau ,$$

where $B_{0}$ is the field value at the beginning of the integration, $A_{c}$ the total area of the coil, and *v* the measured voltage.

The main drawback of this method consists of a magnification of low-frequency noise components that result in a drifting integrated signal. These components can be characterized as a flicker 1/f noise originating from intrinsic sources in the electronic components such as the operational amplifiers’ bias currents and input offset voltage, temperature variations of the electronics, thermocouple voltages along the signal path, and other non-linear effects [6]. In general, the measured voltage *v* can be decomposed in the sum of two terms: the measurand signal $v_{s}$ and an offset term $v_{o}$ superimposed to $v_{s}$, causing drift.

(2) $$v\left(t\right)=v_{s}\left(t\right)+v_{o}\left(t\right).$$

**Figure 1:**
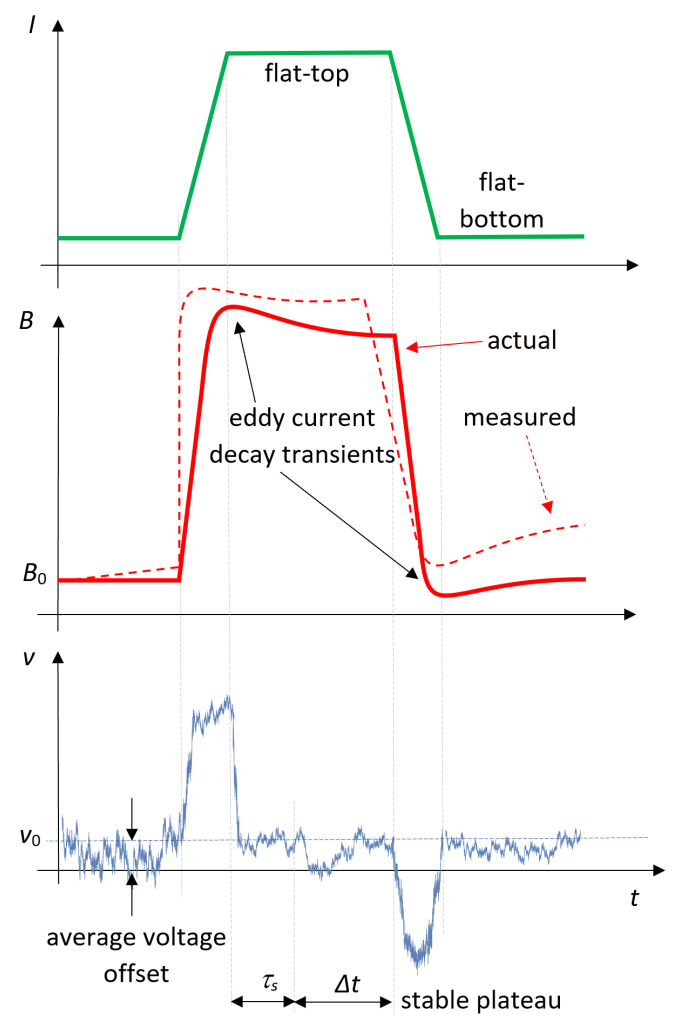
Schematic representation of a synchrotron magnet cycle. The offset voltage $v_{o}$ determines an increasing deviation of the field measurement from the actual value.

Achieving drift-free integration means suppressing or making the integrated $v_{o}$ negligible with respect to the integral of the measurand signal. Figure 1 provides a visual representation of the problem in the domain of synchrotron electromagnets, which are typically operated in a cyclic fashion between a field flat-bottom and a flat-top, where the current is kept constant.

Ideally, the coil voltage signal should be zero on these plateaus; in truth, this is often the case only on their final part, when any eddy currents induced during the preceding ramp have decayed. It can be observed that the voltage offset varies much more slowly than the signal of interest so that in a first approximation, it can be considered as constant during the entire integration period, resulting in a linearly drifting output of the measured field *B* (see Figure 1). This is the principle of the most basic drift correction algorithms.

As voltage integration applies to many different domains such as particle accelerators, nuclear fusion engineering or inertial sensors, and integration’s drift is intrinsically present, a vast literature is available where hardware or software-based drift reduction techniques are presented.

### 1.2. Hardware-Based Techniques

Hardware mitigation of the drift was achieved in [7], where a hybrid analog-digital integrator was proposed to integrate data from accelerometers in earthquake monitoring. The integration was performed using voltage-to-frequency conversion (VFC) implemented in two 12-bit counters, and the drift was corrected by a feedback loop of the integrated signal. The technique presented a good performance on a short time scale (10 s), but it is intrinsically affected by problems of deadband typical of VFCs. In [6], 1/f noise was smoothed in an analog integrator module by adopting different solutions such as guard rings and polypropylene capacitors to reduce the impact of leakage currents. Moreover, a temperature control module was implemented on-board to keep the temperature variations below 0.5 °C, and it suppresses the temperature effects on the bias currents and voltages of the operational amplifiers. In this manner, the integrator drift was kept within 3% on a time scale of 1000 s. Another technique is presented in [8], where a custom Σ-Δ Analog-to-Digital Converter (ADC) using Correlated Double Sampling (CDS) technology and autozero amplifiers was designed with the purpose to mitigate the ADC offset and 1/f noise.

In fusion engineering applications, inductive magnetic field measurements are used for diagnostic monitoring of the magnetic field interacting with the plasma. For this kind of measurement, digital integrators are generally used. The challenging requirement adopted for the electronics design is to keep the drift below 0.12 ppm/s. In [9], the drift was mitigated by using two integrator modules alternatively switched and reset. Another widely used approach [10,11] is the chopping technique, which consists of reversing the polarity of the signal at the input terminals and by integrating the rectified signal, achieving a drift of 0.05 ppm/s.

In particle accelerator applications such as iron-dominated synchrotron magnets, real-time monitoring of the magnetic field is critical for the control of the particle beam, and a very demanding accuracy of the order of a few 10${}^{-5}$ T is required. At CERN, this is realized by using the so-called *B*-train systems where integrator drift correction was implemented by using short integration periods (maximum of a few seconds) combined with reference markers based on Nuclear Magnetic Resonance (NMR) or Ferrimagnetic Resonance (FMR) technology [12,13,14,15]. In this manner, a drift of about 2.3 ppm/s over a time scale of 1–2 s is achieved. Afterward, the drift is zeroed at the beginning of every cycle by resetting the integrator.

All of these hardware-based solutions need custom-made acquisition hardware. Techniques such as CDS or chopping can also be implemented on commercial hardware, but they show unsatisfying performance. Chopping introduces high-frequency harmonics due to switching. These harmonics are filtered due to the limited bandwidth of commercial hardware, resulting in a filtered switching input and a corrupted rectified signal. CDS can amplify drift due to interchannel differences on the same DAQ board. On the other hand, the solution based on reference markers presents the best performance so far (5 ppm repeatability), but it is the most expensive, and the installation of the markers might not always be possible due to mechanical constraints.

### 1.3. Software-Based Techniques

Software algorithms for correcting drift rely on the a priori knowledge of the signal properties and the acquisition chain. For instance, the method of the envelope is used in applications, such as rotating coil measurements, where the signal is known a priori to be periodic [16,17,18]. In [16], in a toroidal ring sample permeameter system, the offset value is updated in correspondence of the flat tops and bottoms of the excitation current waveform, where the induced voltage is expected to be zero. In [19], an a priori identification of the acquisition chain was performed to identify the thermal and electrical dynamic models of the measurement setup and de-trend the output voltage of a toroidal ring-sample permeameter.

Software correction techniques are relatively inexpensive, but they rely on assumptions of the signal properties that might result in artifacts or loss of accuracy.

### 1.4. Proposal

Inertial sensors have in common the problem of integration drift with magnetic sensors. The problem is even more critical in this domain because data from accelerometers have to be integrated twice to measure the position. In this context, sensor data fusion, which combines data from a network of different sensors of different technologies such that the resulting information has lower uncertainty than the single measurement sources individually, is the state-of-the-art approach. In [20], a linear Kalman filter was implemented in an algorithm of 3D orientation detection by combining data from three sensors (a tri-axis accelerometer, a tri-axis gyroscope, and a tri-axis magnetometer). Other examples of sensor fusion applications are reported in [21,22,23,24,25,26,27,28,29], where sensor data fusion was implemented using neural networks or Bayesian inference. However, such an approach of combining data from different sensors has never been adopted so far for inductive magnetic measurements.

This paper shows how sensor data fusion in magnetic measurements can enhance sensing coil accuracy by strongly reducing integrator drift. Two case studies are presented concerning the measurement of the magnetic field inside a dipole magnet. The first one consists of a scenario where data from a Hall probe are combined with data from a sensing coil. The sensor fusion between a Hall sensor and a sensing coil represents an ideal test case because the coil output is the time derivative of the Hall probe output; therefore, sensor fusion can be easily implemented by using a linear first-order Kalman filter. The second one shows a proof-of-concept, where the measurement of the magnet excitation current is used as an inexpensive replacement of the Hall probe. An implementation of the correction algorithm that can be applied either offline, i.e., to post-processing data previously acquired, or online in a real-time measurement context is discussed. In Section 2, the general algorithm, the experimental setup and the two case studies are presented. Section 3 presents experimental results, which are then discussed in Section 4.

## 2. Measurement Method and Data Fusion Algorithm

### 2.1. General Sensor Data Fusion Algorithm

The data fusion algorithm consists in both cases of a linear Kalman filter. Kalman filters are based on a process model describing the evolution of the state at the instant *k* from the one at the instant k−1. In the linear case, the equations of the Kalman filter are in the following form.

(3) $$x_{k}=Fx_{k-1}+Cu_{k}+w_{k},$$

(4) $$z_{k}=Dx_{k}+q_{k}.$$

The first equation is the state-space equation, where x is the vector of the state variables, F is the state-transition matrix, u is the input vector, C is the input-matrix, and w is the process noise vector. The second equation is the measurement equation, where measurement z is affected with noise q. D is the measurement matrix. w and q are assumed to be zero-mean Gaussian noise and possess variance $\sigma_{w}^{2}$ and $\sigma_{q}^{2}$, respectively.

The algorithm consists of two steps: prediction and update. In the prediction phase, the state at the instant *k* is predicted from the state at instant k−1, and the error variance is computed:

(5) $$\begin{array}{c} {\hat{x}}_{k}^{-}=F{\hat{x}}_{k-1}^{+}+Cu_{k} \end{array}$$

(6) $$\begin{array}{c} {\hat{P}}_{k}^{-}=F{\hat{P}}_{k-1}^{+}F^{T}+\sigma_{w,k}^{2} \end{array}$$

where the superscripts + and − indicate posterior and prior, respectively. The hat symbol indicates an estimate. $P_{k}$ is the state error covariance matrix at instant *k* and represents the estimate error. The update step consists in the following:

1. Comparison of prior estimation with and evaluation of the following: $y=z_{k}-D{\hat{x}}_{k}^{-}$;
2. Calculation of the Kalman gain: $K_{k}={\hat{P}}_{k}^{-}D^{T}\left(\sigma_{q,k}^{2}+D{\hat{P}}_{k}^{-}D^{T}\right)$;
3. Update of the state vector estimate: ${\hat{x}}_{k}^{+}={\hat{x}}_{k}^{-}+K_{k}y$;
4. Update of the error covariance matrix: ${\hat{P}}_{k}^{+}=\left(I-K_{k}D\right){\hat{P}}_{k}^{-}$, where I is the identity matrix.

### 2.2. Experimental Setup

**Figure 2:**
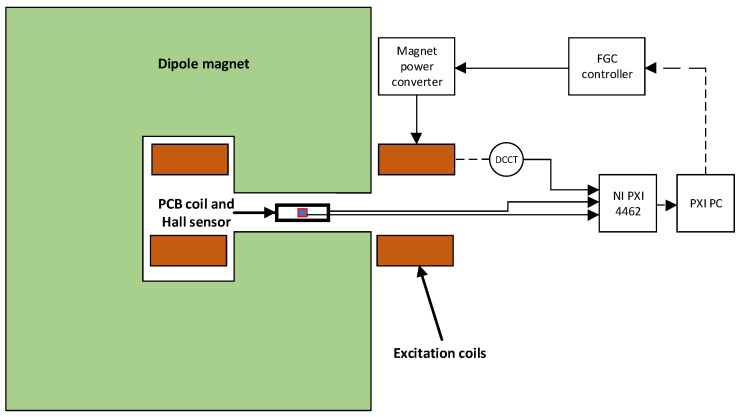
Layout of the measurement system.

**Figure 3:**
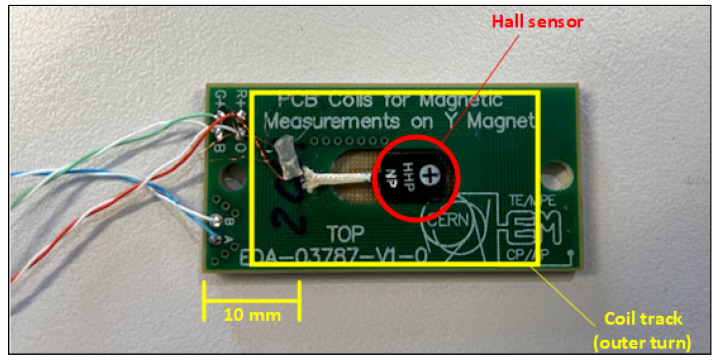
A picture of the combined field sensors. The Hall probe is the black integrated circuit at the center of the PCB.

The two case studies were carried out by using the same experimental setup, as shown in Figure 2, in a C-shaped dipole electromagnet used routinely at CERN for sensor calibration. The sensing coil and the Hall probe were combined on a single printed circuit board (PCB), as shown in Figure 3, to ensure that both are exposed to the same magnetic field, namely the component normal to the PCB, and to enable their simultaneous calibration against an NMR teslameter reference. The sensing coil is physically realized with 161 conducting loops printed on a 16-layer board. The effective area of the coil is $A_{c}=\;$0.059394 m${}^{2}$, calibrated by flipping the coil repeatedly in a 1 T magnetic field. The coil calibration uncertainty is $\sigma_{A}=\;$2.29 $\times \;10^{-6}$ m${}^{2}$, or about 40 ppm in relative terms.

The same sensing coil is used in the second case study, where the Hall probe output is ignored and replaced by the magnet’s current measurement.

The magnet was powered by employing a 120 V/500 A current-controlled power converter controlled by a Function Generator Controller (FGC) [30]. The current was measured using a DC Current Transformer (DCCT) with a current-to-voltage ratio of 100 A/V. The hardware used for the acquisition was a commercial NI DAQ PXI 4462 and it possesses four independent AI channels, each one equipped with a 24-bit Σ-Δ ADC. The Hall probe is an Arepoc HHP-NP 2067, possessing a nominal sensitivity of 223.8 mV/T.

**Figure 4:**
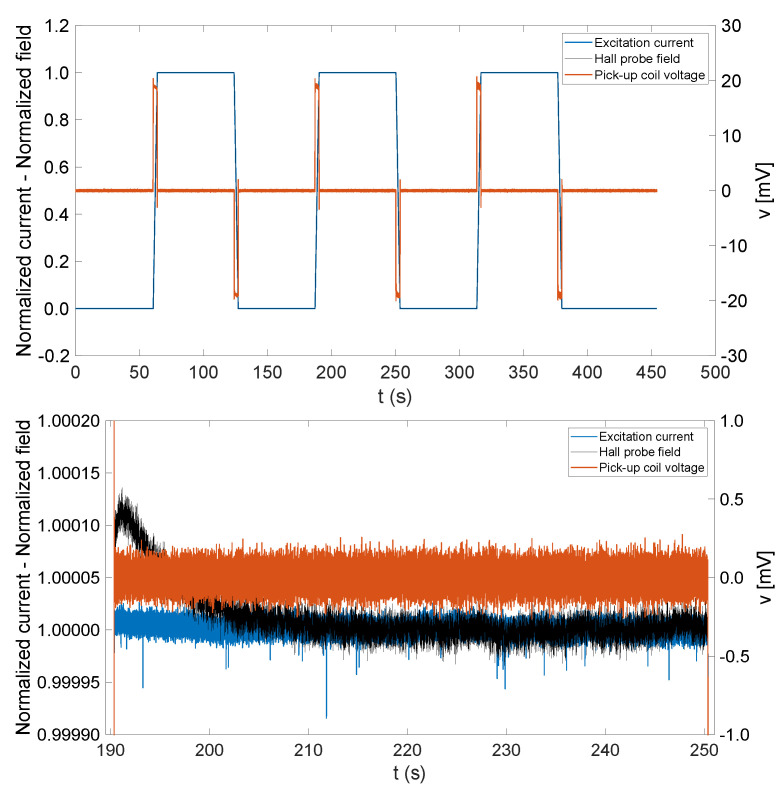
(**Top**) Normalized excitation current, Hall probe field measurements (left vertical axis), and induced voltage in the sensing coil (right vertical axis); (**bottom**) zoom on the central flat-top.

The experiments were performed by ramping the magnet’s current cyclically between 0 and 320 A, corresponding to about 1.012 T. Each ramp is followed by a plateau, where the current level is kept constant for 60 s. Prior to the tests, the magnet was pre-cycled 10 times between 0 and 320 A to bring it on a stable hysteresis loop, wipe out the history of past magnetization, and obtain a repeatable magnet response within 8 ppm, as verified independently via NMR measurements. Three ramp rates were chosen, 3.2 A/s, 32 A/s, and 100 A/s, to validate the proposed approach on different measurement time scales. The signal-to-noise ratio (SNR) of the coil output signal on the ramps scales with the ramp rate and is, therefore, two orders of magnitude higher at 100 A/s, although this does not impact the measured field drift rate appreciably. Figure 4 shows in detail the evolution of the excitation current, the field measured by Hall probe, and the induced voltage on a plateau when a ramp rate of 100 A/s is used. The Hall probe measurement shows clearly an exponential transient with a 120ppm amplitude and a time constant $\tau_{e}\approx 10\;s$, which can be ascribed to the decay of eddy currents arising during the last ramp. As explained in [31], eddy currents in electromagnets can screen or enhance the field locally depending on their distribution in the bulk of the iron yoke, the end region, and in nearby metallic support structures. Indeed, it was experimentally verified that the amplitude and sign of the transient depend strongly on the probe’s position inside the magnet gap. In the figure, the current and field were normalized with respect to the peak value to highlight the dynamic character of the phenomenon. For the purposes of integrator drift estimation and considering the small amplitude of the transient, it is considered in the following that when a time of 2∼3$\tau_{e}$ has elapsed at the end of a current ramp, the stability of the magnetic field should match that of the excitation current.

### 2.3. State-Space Equation

In both case studies the state-space equation is the same and represent the model of a numerical integrator:

(7) $$x_{k}=B_{k}=B_{k-1}+\frac{1}{A_{c}}\frac{\left(v_{k}+v_{k-1}\right)}{2}T_{s},$$

where the following is the case.

(8) $$\begin{array}{c} F=1, \end{array}$$

(9) $$\begin{array}{c} C=\frac{T_{s}}{2A_{c}}, \end{array}$$

(10) $$\begin{array}{c} u_{k}=v_{k}+v_{k-1}. \end{array}$$

$u_{k}$ is expressed as the sum of the voltages at the instants *k* and k−1 because the trapezoidal rule for the integration was adopted. $T_{s}$ is the sampling time. Given the presence of only one state variable, the matrices of the general model are represented by a scalar.

The prior variance of the model and the measurement variances were calculated by propagating uncertainty according to the ISO GUM approach [32] by taking into account type A and type B uncertainty sources. In particular, uncertainty $\sigma_{w,k}$ of the integrated field increment $B_{k}-B_{k-1}$ corresponding to the process model noise in Equation (3) was obtained by propagating uncertainty in Equation (7) and is given by the following:

(11) $$\sigma_{w,k}^{2}=\frac{T_{s}^{2}}{4A_{c}^{2}}\left(\frac{\sigma_{A}^{2}}{A_{c}^{2}}{\left(v_{k}+v_{k-1}\right)}^{2}+\left(\sigma_{v,k}^{2}+\sigma_{v,k-1}^{2}\right)\right),$$

where $\sigma_{v,k}=(2.05+0.003v_{k}$) mV is the uncertainty of the data acquisition system used as specified by the datasheet. In this application, the uncertainty associated with the sampling rate is extremely low and was neglected.

When the Kalman filtering is used, the prior uncertainty of the output value $B_{k}$ is provided by Equation (6) and becomes the following.

(12) $${\hat{P}}_{k}^{-}={\hat{P}}_{k-1}^{+}+\sigma_{w,k}^{2}.$$

### 2.4. Case Study 1—Data Fusion Algorithm of Sensing Coil and Hall Sensor Measurements

When the output of the Hall sensor is used to run the fusion algorithm, the measurement equation is the following:

(13) $$z_{k}=B_{H,k}=B_{k}+q_{k},$$

where $B_{Hk}$ is the measurement performed by the Hall probe, and D=1. The following values were used to run the algorithm:

- Initial model value $B_{0}=$ 2.27 mT, evaluated by Hall probe;
- Hall sensor measurement uncertainty $\sigma_{q,k}=\left(9.02+0.003B_{H,k}\right)$ mT;
- Initial model uncertainty $\sqrt{{\hat{P}}_{0}^{-}}=\sigma_{q,0}=$ 9.02 mT.

### 2.5. Case Study 2—Data Fusion Algorithm of Sensing Coil and Excitation Current Measurements

The relationship between excitation current and the magnetic field is non-linear due to different effects such as ferromagnetic hysteresis, eddy currents, geometry dependencies, etc. Given the size of the sensing coil, geometry dependency can be neglected, and the field-to-current relationship at instant *k* can be modeled as follows:

(14) $$B_{k}=\frac{I_{k}}{g}+f\left(I_{k},I_{k-1},I_{k-2},...\right),$$

where the first term is a first-order approximation of the current-to-field relationship and the second one is the non-linear part, dependent on the past values of the current and its time derivative. Equation (14) is approximated as follows:

(15) $$B_{k}\approx \frac{I_{k}}{g},$$

and, therefore, for this case study, the following is the case:

(16) $$z_{k}=\frac{I_{k}}{g}+q_{k},$$

where D=1/g. This approximation was necessary in order to keep using a linear Kalman filter. *g* is nominally 316 A/T for the magnet under test. The non-linear part was modeled as a disturbance source determining an increase in the uncertainty of *g*. The following values were used:

- Initial model value $B_{0}=$ 0, assuming no information on the remanent magnet field is available;
- Measurement uncertainty $\sigma_{q,k}=\left(0.018+0.006\frac{I_{k}}{g}\right)$ mT;
- Initial model uncertainty $\sqrt{{\hat{P}}_{0}^{-}}=\sigma_{q,0}=$ 0.018 mT. The systematic error arising from not considering the remanent magnet field was not taken into account;
- Current-to-field ratio uncertainty, $\sigma_{g}=$ 1.86 A/T.

## 3. Experimental Results

The result of the two Kalman filters was compared with two feed-forward drift correction algorithms, which take into account the prior knowledge that the field must be constant when the excitation current is constant:

- FFA1—Initial zero-reading average: In this case, the offset voltage $v_{0}$ is estimated by averaging the coil output over a 60 s interval before the actual measurement, when the excitation current is set to zero, which implies that the voltage should be ideally zero. The offset is then assumed to be constant for the entire duration of the measurement, which corresponds to assuming a linear drift.
- FFA2—Online updating algorithm: This algorithm is proposed in [33], and it was adapted to run online. The offset voltage is modeled as a piece-wise constant function, where the estimate is updated periodically by averaging over intervals of duration $\tau_{a}=1$ s once the magnetic field reaches a stable plateau (see Figure 1), after the eddy current transient is fully damped. For the experiments presented in this paper, the duration of the eddy current transient at the end of each current ramp is 20 s at 3.2 A/s, 25 s at 32 A/s, and 30 s at 100 A/s, and the stable plateau duration Δt is, respectively, 40 s, 35 s, and 30 s. In this manner, the offset is assumed to remain constant on ramps and during transients.

**Figure 5:**
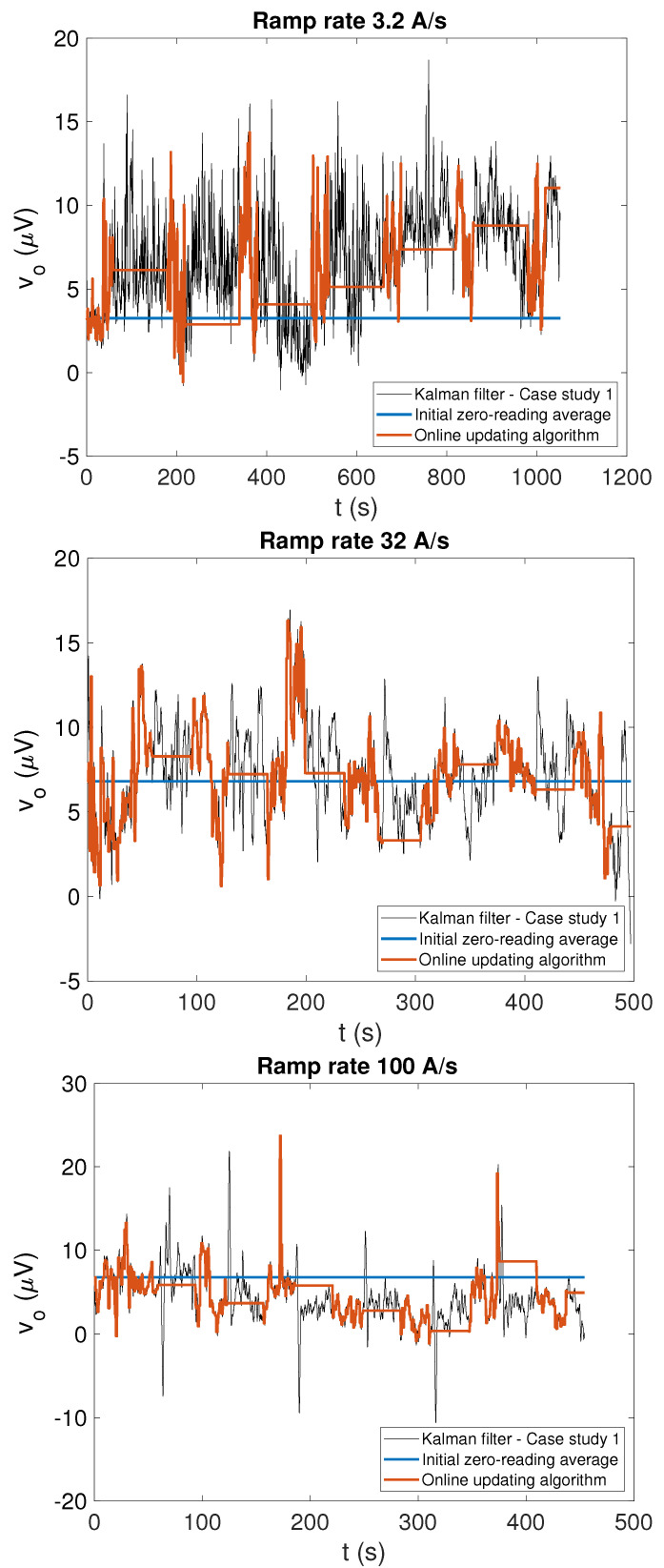
Estimated offset voltage values obtained by Kalman filtering (black), when the field values measured by Hall probe are used in the measurement equation (case study 1) and the two feed-forward offset correction algorithms (FFA1 in blue, FFA2 in orange) plotted for comparison. The corresponding field values obtained by applying the different algorithms can be observed in Figure 7. The flat parts of the FFA2 curve correspond to cycle ramps, where the offset is not updated.

**Figure 6:**
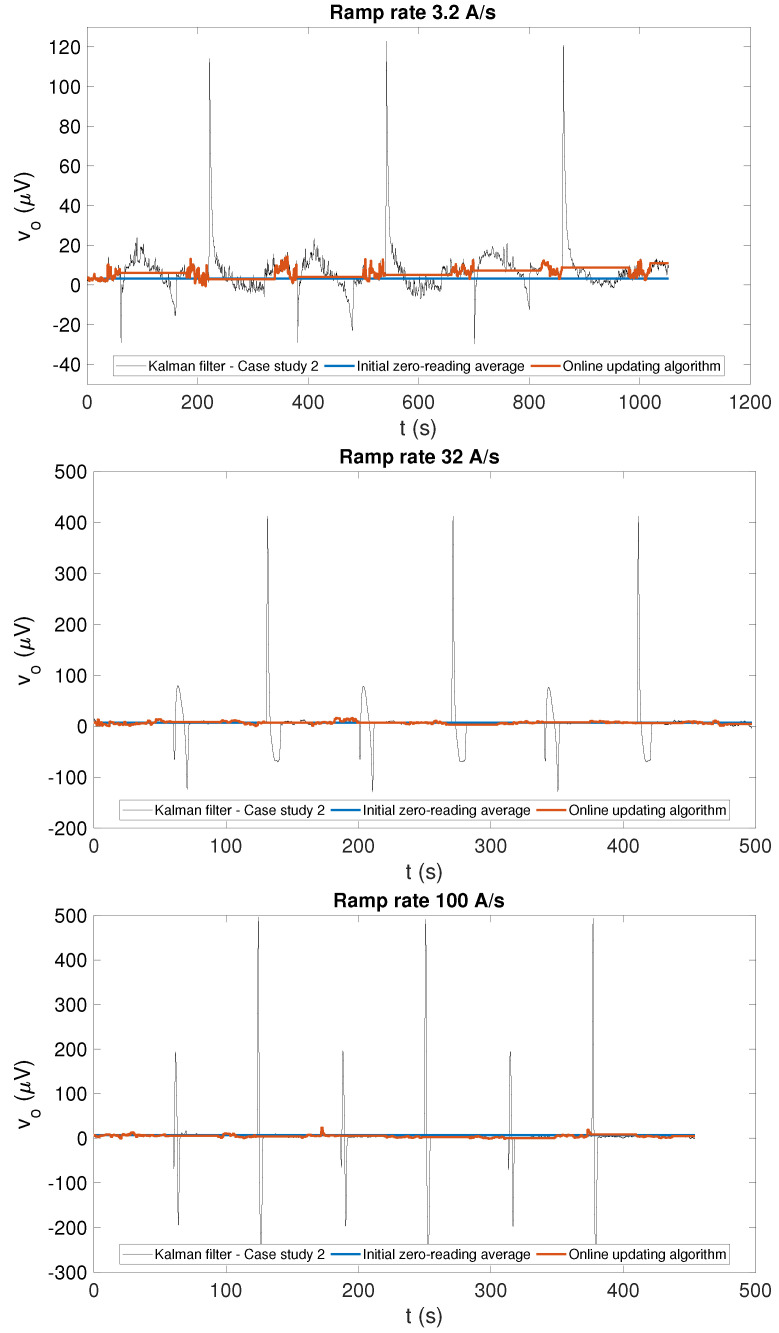
Estimated offset voltage values obtained by Kalman filtering (black), when the field values estimated from the excitation current are used in the measurement equation (case study 2), and the two feed-forward offset correction algorithms (FFA1 in blue, FFA2 in orange), plotted for comparison. The corresponding field values obtained by applying the different algorithms can be seen in Figure 7.

The voltage offset estimated by the four different algorithms is shown for the three tested ramp rates in Figure 5 and Figure 6 for case study 1 and 2, respectively, while a summary of its average and range is provided in Table 1. A moving average over a time window of 1 s was applied to the voltage offset estimated by Kalman filtering to smooth out non-physical variations and obtain a clearer comparison with the output of two feed-forward correction algorithms.

**Figure 7:**
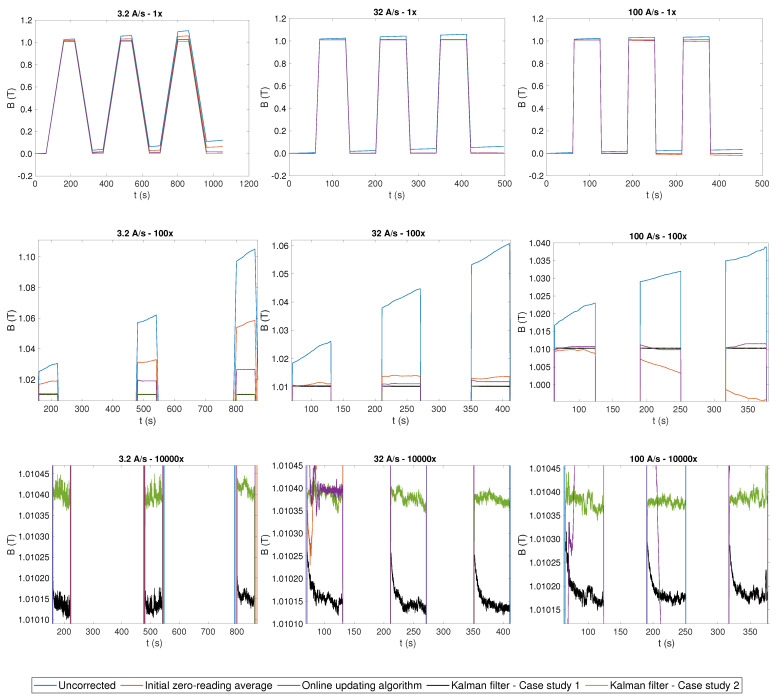
Magnetic field values obtained by Kalman filtering (black and green), when the field values measured by Hall probe are used in the measurement equation (case study 1), when the field values are estimated from the excitation current (case study 2), field values obtained by using the two feed-forward offset correction algorithms (orange and purple), and field values when no drift correction is applied (blue). From top to bottom: 1×, 100×, and 10,000× magnification. From left to right: 3.2 A/s, 32 A/s, and 100 A/s.

**Figure 8:**
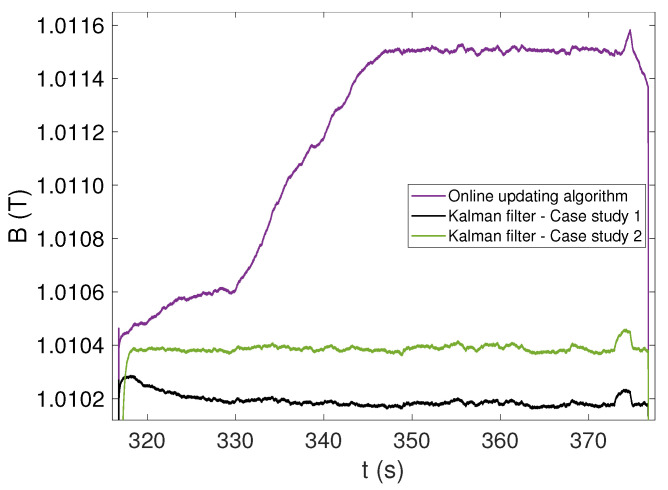
Comparison of case study 1 and 2 algorithms (black and green, respectively) with the algorithm FFA2 on the last flat-top, when a ramp rate of 100 A/s is applied.

The uncorrected integrated field is plotted in Figure 7 as a baseline, alongside the results obtained by applying the two feed-forward correction algorithms FFA1 and FFA2 and the two Kalman filters CS1 and CS2. The three columns of plots represent different ramp rates, while the three rows of plots show increasing magnification levels to highlight better the flatness of the plateaus achieved by different correction algorithms. A zoomed-in view of a detailed comparison between CS1 and CS2 is shown in Figure 8.

Table 2 reports a summary of the measurement results by comparing the two case studies and the two feed-forward estimation algorithms for three different ramp rates. A performance indicator, $\delta_{G}$, was defined to quantify how much the measured field globally drifts during the measurement relative to its initial value:

(17) $$\delta_{G}=\left|\frac{\frac{dB}{dt}}{B_{B}}\right|=\left|\frac{B_{F}-B_{B}}{TB_{B}}\right|,$$

where $B_{B}$ is the field at the beginning of the first flat-top, $B_{F}$ is the field at the end of the last flat-top, *T* is the time interval between these two reference values, and $\frac{dB}{dt}$ is the global field drift.

The performance of the correction was also assessed in terms of the total uncertainty of the measured field, $\sigma_{B,k}$, defined by the following:

(18) $$\sigma_{B,k}=\sqrt{P_{k}}+\left(B_{k}-B_{k}^{CS1}\right),$$

where for CS1 and CS2, $P_{k}={\hat{P}}_{k}^{+}$, because the a posteriori variance estimated by the Kalman filter is used. For the uncorrected integral and the two algorithms FFA1 and FFA2, $P_{k}={\hat{P}}_{k}^{-}$ is used. This means that the uncertainty is not updated at each time step as in CS1 and CS2, and the uncertainty contribution provided by process model noise described in Equation (11) accumulated and increased with time. Considering that the uncertainty calculation only considers a stochastic contribution, a systematic component representing the drift contribution was added. This component was estimated as the difference between the current magnetic field value and the one obtained by the Hall probe-based Kalman filter, considered as reference.

Each algorithm can be characterized by considering the final value on the last flat-top, $\sigma_{B,end}$. A coverage factor of 2 was applied to obtain the expanded uncertainty corresponding to a 95% level-of-confidence interval (Table 3). The values of *σ_B,end_* are reported in Table 3.

## 4. Discussion

At the ramp rate of 32 A/s, the uncorrected field integral drifts globally by about 120 ppm/s or, equivalently, 120μT/s, resulting in a 6% error over the entire duration of the measurement. Similarly to all other test results, this error does not appear to be correlated with the ramp rate; instead, the voltage offset is observed to fluctuate slowly and randomly with time.

All four algorithms provide a roughly comparable voltage offset estimate ranging between −3 and 22 μV. The simplest algorithm, FFA1, taking information only from a brief initial stretch of data, provides a single estimated value that is generally inconsistent with the mean values obtained with the other algorithms and cannot follow precisely the evolution of low-frequency noise components. In terms of global drift, its error reduction ranges unpredictably from a mere 10% at 100 A/s to a factor of 15 at 32 A/s. However, even in the best case, the measured field’s uncertainty on the plateaus is poor and in the order of tens of mT.

The online updating algorithm, FFA2, instead provides an average offset estimate in line within 10% of the Kalman filters. This algorithm results in a much better correction, especially on the plateaus, after the eddy current decay transient, where the offset can be updated regularly; the total uncertainty improves by a factor up to 16 with respect to the uncorrected integral. However, a visible accumulated drift remains between plateaus due to poor estimation on the ramps and during the eddy current decay transient. The drift increases from 2.95 ppm/s at 100 A/s to 55.47 ppm/s at 3.2 A/s, which is due mainly to the longer interval between successive plateaus. At this ramp rate, FFA2 provides no significant improvement with respect to FFA1.

The Kalman filtering algorithm based on the Hall probe measurements (CS1) does not suffer from any of these drawbacks. At all ramp rates, the global drift is better than 0.04 ppm/s, representing an improvement of three orders of magnitude compared to the uncorrected integral. The total uncertainty on the stable part of the plateaus is consistently 65 μT. Such excellent performance is due to the ability of the filter to capture fluctuations of the offset also on the current ramps and during the eddy current transients, when the estimate provided by FFA2 cannot be updated.

The Kalman filtering algorithm based on excitation current measurements (CS2) provides voltage offset estimates on the plateaus within 0.5 μV from CS1 and FFA2, which reinforces confidence in these results. However, large voltage spikes, increasing with ramp rate and reaching up to about 500 μV at 100 A/s, can be observed in correspondences of the ramps in Figure 6. These spikes are nonphysical artifacts because Equation (16), in contrast with Equation (13), does not contain any information about the dynamics of the magnetic field. This results in a systematic error in the magnetic field on the plateaus, which is clearly visible in the bottom row of Figure 7, providing a total uncertainty as high as 667μT, i.e., one order of magnitude higher with respect to CS1. Nonetheless, the performance in terms of drift cancellation is very similar to CS1, and the field on the plateaus is stabilized below 0.1 ppm/s, which is also three orders of magnitude better than what is achieved by the two feed-forward algorithms.

## 5. Summary and Conclusions

In this article, a data fusion approach was proposed to improve the correction of integrator drift in inductive magnetic measurement applications. The approach is based on Kalman filtering, where the model output of the integrator is continuously updated by using data from another sensor, representing directly or indirectly the magnetic field. The first proposed algorithm (CS1) combines the data from a sensing coil and a Hall sensor. The second algorithm (CS2) combines coil data with a field estimation calculated from the excitation current, which may be a useful alternative option whenever an additional magnetic field sensor is not practical. Both algorithms were tested in an experimental setup, representing the conditions typically found in accelerator beamline electromagnets.

Both algorithms effectively suppress the drift by stabilizing the measured field on the final part of magnetic cycle plateaus within 20 ppm on time scales up to 1100 s, closely comparable with the rated stability of the power supply used. In terms of globally observed drift, using a Hall probe allows an improvement of three orders of magnitude down to 0.03 ppm/s compared to an uncorrected drift up to 112 ppm/s. Replacing the Hall probe with the current measurement achieves similar results in terms of global drift reduction; however, systematic errors of the corrected plateau levels one order of magnitude higher up to about 700 ppm. At any rate, the performance of both Kalman filters is two to three orders of magnitude better than simple algorithms based on averaging and subtracting the coil voltage output, such as the ones used routinely in fixed-coil applications at CERN.

The proposed approach has a potentially vast range of applications for improving the accuracy of the characterization and modeling of magnetic devices. In the two case studies reported, Kalman filtering was carried out offline, i.e., in the post-processing phase; however, the algorithm is well adapted to real-time implementation, and it could be potentially very useful for magnetic field control applications in fusion devices and particle accelerators. Some of the aspects on which further work is planned include extending the method to long, slender induction coils such as the ones used to measure the integral field produced by accelerator magnets and developing suitable formulations for non-linear current-to-field relationships (Equation (14)) in order to improve accuracy when only the excitation current can be used. In particular, preliminary investigations showed promising results with respect to the application of recurrent neural networks [34] or more complex neural architectures [35] in terms of representing dynamic magnetic phenomena such as eddy currents and hysteresis. The use of high-precision measurements obtained by the Hall probe-based Kalman filter in a controlled laboratory setting is currently being considered for training a network that will then be used to supplement simple current measurement in a real-time operational context.

## Figures and Tables

**Table 1:** Mean and range of the estimated voltage offset $v_{0}$.

	Ramp Rate						Unit
	3.2 A/s		32 A/s		100 A/s		
	Mean	Range	Mean	Range	Mean	Range	
FFA1	3.3	-	6.8	-	6.8	-	μV
FFA2	6.0	[−1, 14]	6.9	[1, 16]	4.6	[−1, 24]	μV
CS1	6.9	[−1, 19]	7.2	[−3, 17]	4.4	[−11, 24]	μV
CS2	6.9	[−29, 122]	7.2	[−129, 412]	4.4	[−250, 496]	μV

**Table 2:** Global relative drift values expressed by means of the parameter $\delta_{G}$.

	Ramp Rate			Unit
	3.2 A/s	32 A/s	100 A/s	
Uncorrected integral	112.14	119.98	59.90	ppm/s
FFA1—Initial zero-reading average	59.25	8.03	53.02	ppm/s
FFA2—Online updating algorithm	55.47	5.05	2.95	ppm/s
CS1—Kalman filter with Hall probe	0.03	0.04	0.03	ppm/s
CS2—Kalman filter with excitation current	0.02	0.03	0.08	ppm/s

**Table 3:** Total uncertainty values $\sigma_{B,end}$ defined according to Equation (18).

	Ramp Rate			Unit
	3.2 A/s	32 A/s	100 A/s	
Uncorrected integral	194.48	104.23	60.34	mT
FFA1—Initial zero-reading average	100.25	9.38	25.97	mT
FFA2—Online updating algorithm	37.57	6.53	5.53	mT
CS1—Kalman filter with Hall probe	65	65	65	μT
CS2—Kalman filter with excitation current	667	533	482	μT

## Data Availability

The data that support the findings of this study are available from the authors, upon reasonable request.

## Abbreviations

The following abbreviations are used in this manuscript:

VFC	Voltage-to-Frequency Converters
CDS	Correlated Double Sampling
ADC	Analog-to-Digital Converter
CERN	European Organization for Nuclear Research
NMR	Nuclear Magnetic Resonance
FMR	Ferrimagnetic Resonance
ELENA	Extra-Low ENergy Antiproton ring
PCB	Printed Circuit Board
FGC	Function Generator Controller
DCCT	DC Current Transformer
GUM	Guide to the Uncertainty of Measurements
NI	National Instruments
DAQ	Digital Acquisition
SNR	Signal-to-Noise Ratio
